# Recent advances in the clinical management of intoxication by five heavy metals: Mercury, lead, chromium, cadmium and arsenic

**DOI:** 10.1016/j.heliyon.2025.e42696

**Published:** 2025-02-14

**Authors:** Mahdi Balali-Mood, Nastaran Eizadi-Mood, Hossein Hassanian-Moghaddam, Leila Etemad, Mohammad Moshiri, Maryam Vahabzadeh, Mahmood Sadeghi

**Affiliations:** a Medical Toxicology and Drug Abuse Research Center, Birjand University of Medical Sciences, Birjand, Iran; b Department of Clinical Toxicology, School of Medicine, Isfahan Clinical Toxicology Research Center, Isfahan University of Medical Sciences, Isfahan, Iran; c Social Determinants of Health Research Center, Shahid Beheshti University of Medical Sciences, Tehran, Iran; d Royal Perth Bentley Group, Next Step Drug and Alcohol Services, Perth, Australia; e Medical Toxicology Research Center, Mashhad University of Medical Sciences, Mashhad, Iran; f Pharmaceutical Research Center, Pharmaceutical Technology Institute, Mashhad University of Medical Sciences, Mashhad, Iran; g Department of Pharmaceutical and Food Control, School of Pharmacy, Mashhad University of Medical Sciences, Mashhad, Iran

**Keywords:** Heavy metals, Toxic heavy metals, Complications, Poisoning, Treatment, Chelation therapy, Chelating agents, Chelators, Toxic metals, Antidote, Intoxication, Antioxidant

## Abstract

Metals have been used for many centuries, but their nutritional and toxic effects have been investigated since the last century. The common toxic heavy metals (THM) include mercury, lead, chromium cadmium, and arsenic. As human exposure to THM increasingly causes systemic and organ complications, it seems required to review the recent advances of treatment of the toxic metals.

Despite the current knowledge of the hazards of heavy metals, there is still high incidents of their poisonings particularly in developing countries. In this review, after an introduction, we briefly describe the routes of exposure, clinical features and prognosis of each metal poisoning. Then, review the different treatments for each metal with particular attention to recent advances in the treatment of both acute and chronic poisonings. The main antidotes for all THM are still chelating agents, but new agents were developed over the past decades and have been used successfully for the THM poisonings. Dimercaptosuccinic acid (DMSA) known as succimer has been prescribed as a safe oral chelator in lead poisoning. Similarly, dimercapto-propanesulfonic acid (DMPS) has also revealed fewer side effects than the old chelating agents. The two are currently gaining increased acceptance among clinical toxicologists. However, there is no specific antidote for mercury poisoning. Dimercaprol is almost no longer used as an antidote of choice in the treatment of chronic THM poisoning.

Comparison of clinical management of intoxication by the five heavy metals reveals similar treatment strategies. On the other hand, some of them require specific interventions to reduce the toxicity. Because of drawbacks in the application of commonly known chelating agents, treatment with bioactive compounds which have antioxidant and anti-inflammatory properties has been the subject of much interest in recent research. However, despite the promising results observed in experimental animals, clinical trials on their clinical therapeutic benefits have not been yet successful and need further studies to determine their efficacy and safety in humans. Development of less toxic chelating agents are still under investigations. Moreover, the development of orally administrable chelating agents for home health care would likely be of great interest for future research.

## 1 Introduction

Heavy metals are widespread toxicants that humans have come in contact with since last centuries. Human exposure to these metals can be arisen due to natural or anthropogenic activities. While their use is beneficial for a facilitated life (Table 1), environmental exposure can be hazardous and cause toxic adverse effects [[1], [2], [3]]. However, some metals that are considered as essential micronutrients are responsible for the important biochemical processes in the body. Copper is involved in the enzymes superoxide dismutase (SOD), cytochrome oxidase, alcohol dehydrogenase (ALD), alkaline phosphatase (ALP), ascorbate oxidase, tyrosinase and lysyl oxidase [[4], [5], [6]]. Copper also plays a crucial role in iron absorption and heme biosynthesis [6]. Cr^3+^ is a component of the glucose tolerance factor and plays a decisive role in insulin metabolism [7]. Selenium is the prosthetic group of glutathione peroxidase. Other metals like iron, zinc, magnesium, sodium, potassium and calcium are essential for the body [8]. Nevertheless, Cr^6+^ and the high levels the essential and nutrient metals are toxic and should be monitored as clinically indicated [9]. In this review, we considered five common toxic heavy metals (THM); including Mercury (Hg), lead (Pb), chromium (Cr), cadmium (Cd) and arsenic (As).

**Table 1:** Common THM compounds, their chemical formula and applications.

Chromium (Cr) compounds	Chemical Formula	Uses
Barium chromate (Cr^6+^)	BaCrO_4_	Anticorrosive, safety matches, paint pigment
Calcium chromate (Cr^6+^)	CaCrO_4_	Batteries, metallurgy
Chromic acid (Cr^6+^)	H_2_CrO_4_	Oxidizer, electroplating,
Lead chromate (Cr^6+^)	PbCrO_4_	Paints and dye Yellow pigment
Potassium dichromate (Cr^6+^)	K_2_Cr_2_O_7_	Leather tanning, oxidizer of organic compounds, porcelain painting
Chromic chloride (Cr^3+^)	CrCl_3_	Total parenteral nutrition
Chromium picolinate (Cr^3+^)	C_18_H_12_CrN_3_O_6_	Nutritional supplement
Chromic fluoride (Cr^3+^)	CrF_3_	Protecting against moth for wool and silk, fixing material in dye industry
Chromic oxide (Cr^3+^)	Cr_2_O_3_	Metal plating, wood treatment
Chromite ore (Cr^3+^)	FeCr_2_O_4_	Water tower treatment

Cadmium (Cd) compounds	Chemical Formula	Uses

Cadmium carbonate	CdCO_3_	Paints and dye pigment
Cadmium chromate	CdCRO_4_	Chemical manufacturing
Cadmium sulfide	CdS	Solar cells, light emitting diodes
Cadmium oxide	CdO	Paints and dye pigment
Cadmium telluride	CdTe	Solar cells

Mercury (Hg) compounds	Chemical Formula	Uses

Mercury acetate	Hg(CH_3_CO_2_)_2_	Chemical and pharmaceutical manufacturing
Mercury chloride	HgCl_2_	Disinfectant, photography
Methylmercury chloride	CH_3_HgCl	Chemical Manufacturing
Phenylmercury acetate	CH3CO_2_HgC_6_H_5_	Used in pesticides

Lead (Pb) compounds	Chemical Formula	Uses

Lead acetate	Pb(CH_3_CO_2_)_2_	Chemical manufacturing, pigment production
Lead nitrate	Pb(NO_3_)_2_	Manufacture of explosives, pigment production, chemical manufacturing
Lead chloride	PbCl_2_	Chemical manufacturing, ceramics manufacture
Lead thiocyanate	Pb(SCN)_2_	Manufacture of explosives
Lead oxides (lead tetroxide and lead dioxide)	Pb_3_O_4_ and PbO_2_	Battery and pigment manufacturing

Arsenic (As) compounds	Chemical Formula	Uses

Sodium arsenite	NaAsO_2_	Used in pesticides
Arsine	AsH_3_	Chemical warfare agent, metals refining industries
Arsenates of sodium, calcium, potassium, lead	AsO_4_^−^	Disinfectant, paper industries

THM begin to accumulate in the body following a close contact in the environment via either chronic or acute exposure. Under these conditions and by the accumulation of THM in the body, the harmful effects of their accumulation appear. Thus, millions of children and adults might be affected around the world [10]. Adverse effects of THM may cause loss of important nutritional minerals, disruption of normal physiological activities due to interference/inhibition in enzyme function, damage to the central nervous system (CNS), hematological complications, occurrence of delayed pain and emerging of cancers [11]. Prevention of THM exposure is the first measure of treatment that must be taken. For those intoxicated by THM, a series of therapeutic measures should be performed to lower the blood/serum level of the toxic elements. These can include combatting dietary deficiencies, applying symptomatic treatment and supportive care, and prescribing appropriate medications as required [10]. Chelators are well known pharmaceutical antidotes for the treatment of THM that are being widely used in this regard. While some chelating agents are used for more than one THM, the use of specific chelator for the treatment of poisoning by some THM is advocated. In this study, we aimed to review the recent advances in treatment of poisoning related to the five important and commonly used THM.

## 2 Methods

This narrative review gives a comprehensive context and a specific overview of the present research data on recent advances on the clinical management of the five THM which are of great public health significance. The common therapy of these metals with specific focus on the new drug treatment in recent years for moderate to severe THM poisonings was reviewed. The aim of the present study was to discuss and compare the data on treatment protocols from the primary scientific literature databases including Scopus, PubMed, Web of Science (ISI) and Google Scholar. The search terms were included “treatment”,” management”, “therapy”, “chelators”, “chelating agents”, “antidotes”, “mercury”, “lead”, “chromium”, “cadmium”, “arsenic”, “toxicity”, “poisoning” and “intoxication”. The literature was searched for both animal and human studies involving methods for treatment related to the five THM and any related possible therapies. Unpublished data and letter to the editor were excluded. Case reports, conference papers, and books were not included into our study. Only English articles were included. We preferred to search and review the articles published within the last 20 years. Symptomatic treatment, supportive care, antidotal therapy, and chelating therapy for each metal were extracted from the literature. The similarities and differences of these treatments were compared.

Each of the five THM was drafted by one of the authors (numbers 2–6) who had vast experience in clinical management of THM poisoning. Each draft was first reviewed by the first and corresponding authors and revised by the drafters. Introduction, methods, general discussion and abstract were prepared by the first and corresponding authors and draft of the full papers after several revisions were finally reviewed by the all authors. The first and corresponding authors made the final version for submission.

## 3 THM

### 3.1 Mercury (Hg)

#### 3.1.1 Size of the problem and preventive measures

There are relatively large amounts of vapors of various mercury compounds in the atmosphere mainly due to human activities. They are always in rotation and exchange with high amounts of mercury in the water and soil of different regions of the earth. Therefore, the global anthropogenic Hg emission was estimated more than 1900 metric tons in 1995 [12,13]. Due to above-mentioned points and the many uses of mercury in today's life and industry (which is newly a little more limited), getting mercury from the environment is inevitable and thus, the absolute prevention of mercury pollution seems impossible. On the other hand, mercury bioaccumulation in humans can cause adverse health problems [14,15]. Therefore, prevention of mercury exposure in any way can be very helpful in this regard. However, our exposure to Hg happens mainly through the consumption of MeHg contaminated fish [13,16,17]. But practical measures can be implemented in these fields which include:

I. Public education (especially for children) can play an important role in preventing mercury contamination [18]. Trying not to pollute the environment (water, soil, air and living environment in general) with mercury should be the main focus of these trainings. In addition, learning the methods of cleaning the environment from mercury contamination should be the main focus of specialized training in related professions. Training of the workers in the industries that Hg is used e.g. fluorescent bolb plants is of great importance. They should use protective devices, particularly mask with special Hg filter. The workers should learn how to avoid metal mercury exposure and how to clean Hg in the work environment in case of contamination. They should learn how to prevent their own contamination and minimize the possibility of contamination of the work environment and other colleagues. The necessary prevention facilities of Hg exposure in the workplace should be performed [19].
II. In recent years to avoid Hg exposure, new alternative technologies were used to replace the old methods. For instance, Hg free digital pressure gauges, and Hg-free agricultural pesticides and fungicides have been applied. Prohibiting of amalgam which contained around 50 % Hg in restorative dentistry and using composite instead is another example. According to the World Health Organization (WHO), the main exposure to elemental mercury is from dental amalgams [14,15,20].
III. The use of inorganic mercury in skin lightning products can have diverse effects. Mercurous (Hg^+^) and mercuric (Hg^2+^) salts are common forms of inorganic Hg human exposure. Depending the exposed Hg amount, from no symptoms to severe signs may occur [[21], [22], [23], [24], [25], [26]]. Therefore, producing and encouraging consumers to use Hg-free cosmetics should be considered.
IV. The consumption of food grown in artificial environments such as farmed fish can be very effective in reducing the intake and accumulation of mercury in the body especially when precise scientific methods are used to grow them.

Oxidative stress is an important molecular mechanism of Hg toxicity. Hg may induce oxidative stress through several mechanisms including ROS generation, GSH depletion, alteration of antioxidant enzymes, mitochondrial dysfunction, and inflammatory responses. Vascular endothelial growth factor (VEGF) upregulation in endothelial cells following MeHg exposure can induce endothelial inflammation. Such endothelial/vascular dysfunction can increase the risk of cardiovascular disease [27]. Oxidative stress resulting from inorganic Hg deposits in the brain via the increase of lipoperoxidation, MDA and nitrite concentration, and a decrease of the total antioxidant capacity leads to fine motor dysfunction linked to imbalance of redox status in the cortex, apoptosis, and cellular death [28]. An *in vitro* treatment of human embryonic kidney epithelial cell line with HgCl_2_ showed a decrease in cell viability and oxidative stress mediated by the mitochondrial disorder and upregulation mitochondrial fission at the cellular level [29].

#### 3.1.2 Acute mercury poisoning

Oral ingestion of Hg^0^ rarely causes acute toxic effects, it is absorbed as low as 0.01 % of the dose in the gastrointestinal (GI) tract. Ingestion of a single dose of elemental mercury presents a negligible risk of toxicity [19]. Acute intoxication is commonly linked to the inhalation of Hg^0^ or ingestion of inorganic mercury. Acute Hg inhalation can lead to pulmonary dysfunction and other organ disorders. Emerging signs and symptoms are fever, shortness of breath, chills, metallic taste and pleuritic chest pain. Stomatitis, confusion, lethargy and vomiting are other possible symptoms. Pulmonary complications of Hg inhalation may progress to interstitial emphysema, pneumatocele, pneumothorax, pneumomediastinum and interstitial fibrosis. Acute poisoning of inorganic mercury or mercuric salts is usually the cause of oral ingestion. The acute signs and symptoms of toxicity caused by inorganic mercury or mercuric salt are primarily due to their corrosive properties. The acute presentation can lead to various signs, such as ashen-gray mucous membranes, hematochezia (bloody stool), severe abdominal pain, vomiting and hypovolemic shock. Systemic effects usually start several hours after ingestion, including mucosal inflammation, metallic taste, gingival irritation, renal tubular necrosis and loosening of teeth [19,[30], [31], [32], [33]]

#### 3.1.3 Chronic mercury poisoning

Chronic intoxication is more common from organic Hg exposures. Chronic exposure can also result from delayed occupational exposure to Hg^0^ that can be transformed into the inorganic form in the body. The other long-term exposures include topical application of mercurial salts and the chronic use of diuretics or cathartics containing Hg. Chronic Hg exposure can cause various renal, neurological, psychological and cutaneous disorders. Non-specific symptoms include anorexia, weight loss, fatigue and muscular weakness that could be indicative of multiple diseases [30]. Chronic poisoning markedly affects the central nervous system and the kidneys. Tremors are present in the facial muscles, eyelids and late in the limbs. Irritability, fears, shyness, loss of confidence are commonly observed. Frank nephrotic syndrome can be developed following chronic Hg exposure. The most common indication of renal disorders is proteinuria-reflecting glomerular damage [19,[30], [31], [32], [33]].

#### 3.1.4 Prognosis for mercury poisoning

Like all other toxicities, the prognosis of poisoning depends on various factors. a good prognosis may be achieved by early detection. Besides, the prognosis relies on the duration of exposure, Hg concentration and the severity of clinical manifestations [26]. The prognosis for Hg exposure is highly variable and it depends on the level and duration of exposure. High exposure can cause coma and death. Other minor symptoms may not be diagnosed and resolve over time. Neurologic disorders which can be delayed in the presentation may last for decades without diagnosing of Hg poisoning, unless the physician considered the history of Hg exposure. Fetus and children are highly susceptible to Hg exposure. It may produce severe toxicity leading to death or permanent neurologic deficits such as mental retardation [34]. In nephrotic syndrome following the Hg toxicity, using chelating agents D-penicillamine and 2,3-dimercapto-1-propanesulfonic acid (unithiol, DMPS) alone or in combination with other treatments has been shown to have a good prognosis with improvement of clinical manifestations and lab parameters [26,[35], [36], [37], [38]].

#### 3.1.5 Severity classification (mild, moderate and severe)

There is no distinctive categorization for severity classification in this element. Hg concentration in whole blood below 20 μg/L is considered normal. This can rise to 35 μg/L after long-term exposure to Hg vapor. Hair analysis can present Hg exposure that has occurred over an extended period of time. Hair Hg concentration is a biomarker of long-term exposure to MeHg. Severe cases may be seen after acute exposure to methylmercury particularly in infants or children with neurological and GI symptoms, respiratory distress, and dermatitis mainly. The hair Hg concentration of more than 2400 mg/kg shall be expected. While pregnant mothers may have little or no manifestation of MeHg intoxication, exposed infants may face decreased birth weight and muscle tone, seizure, developmental delay, severe spasticity, blindness and deafness. In general, hair Hg concentration does not exceed 10 mg/kg. While in moderate mercury poisoning, the concentration level ranges between 200 and 800 mg/kg. WHO recommends MeHg evaluation in pregnant women's hair and warn that levels exceeding 10 ppm can elevate the risk of fetal neurological defects [39]. The health impact of Hg exposure is primarily focused on chronic, low or moderate grade exposure of MeHg.

#### 3.1.6 Common treatment of mercury intoxication

The first consideration in treatment of acute Hg exposure generally is removal of the patient from further exposure. Support of respiratory and cardiovascular function is also conducted. There is no specific antidote for mercury, but chelation therapy is favored in symptomatic patients. However, the effectiveness of therapy with chelating agents is uncertain in severely poisoned patients. Moreover, indications for the therapy have not been completely defined. Dimercaprol (British Anti-Lewisite, BAL), D-penicillamine, DMPS and succimer (meso-2,3- dimercaptosuccinic acid, DMSA) are among the chelating agents that can be utilized for the management of acute inorganic (Hg^2+^) and metallic (Hg^0^) mercury intoxication. The U.S. FDA has not approved any chelators for methylmercury or ethylmercury. BAL is contraindicated for the management of MeHg intoxication [39]. N-acetyl-D,L-penicillamine is an analog of D-penicillamine and has shown to be effective in Hg elimination from the body. The chelating drug is taken orally. It was claimed that N-acetyl-penicillamine poses similar efficacy and less side effects than D-penicillamine for the treatment of Hg poisoning [40,41].

#### 3.1.7 Recent advances in the treatment of mercury intoxication

Shenkang, is a Chinese herbal medication which has been utilized in the clinical management of chronic kidney disease (CKD). The herb has exhibited therapeutic effects in Hg kidney sequalae [42]. Calcium carbonate, garlic tablet and N-acetyl cysteine (NAC) also gain popularity in some studies. Clinical trials are needed to study the effectiveness of new remedies [[43], [44], [45]]. Selenium may elevate distribution of Hg away from target tissue and increase Hg removal. Selenomethionine and sodium selenite are among selenium supplements being used for the management of Hg intoxication. Selenium can antagonize Hg at the molecular level via a few possible pathways; a more plausible one including Se-aided demethylation of MeHg [46]. NAC has shown the ability to bind with Hg. Moreover, NAC is a GSH precursor that can increase GSH production. GSH and many thiol (sulfhydryl, −SH) groups play a key role in defense mechanism against Hg toxicity [47]. Alpha-lipoic acid (α-LA), an organo-sulfur compound, can chelate Hg and some other THM by having two thiols in the structure. α-LA as a chelating agent that is able to penetrate the blood brain barrier (BBB) while other chelators such as succimer and DMPS cannot access to the BBB. α-LA can also regenerate antioxidants and hinder the free radical reactions [48]. Flavonoids has shown protective effect against kidney and liver injuries resulting from mercury chloride [49]. Myricetin, a flavonoid compound present in medicinal plants, has shown protective effects against mitochondrial dysfunction induced by MeHg *in vitro* by blocking oxidative stress and regulating inflammation [50].

To summarize, the treatment of Hg poisoning is an expanding field which is continually evolving. Many promising supplements according to recent studies like Shenkang, NAC, α-LA, and selenium show potential therapeutic applications. Clinical trials are indeed essential to validate their efficacy and establish effective treatment measures.

### 3.2 Lead (Pb)

#### 3.2.1 Size of the problem and preventive measures against lead exposure

Exposure to lead and subsequent toxicity in humans occurs via ingestion, inhalation, dermal, and ocular contact; mainly by leaded paint and gasoline, adulterated opium and occupational environment [51]. According to the Occupational Safety and Health Administration (OSHA), the permissible exposure limit (PEL) for Pb is 50 μg per cubic meter of air on average over an 8-h shift [52]. One important source of Lead exposure in opium addicts is adulterated opium, which is now a matter of concern in Iran and other Middle Eastern countries that requires more governmental vigilance [51]. To reduce occupational exposure to Pb, adequate ventilation must be assured along with using personal protective equipment (particulate masks, gloves and goggles), avoiding dust formation and breathing vapors, and inspection for any leakage or spillage. Generally, the main step to prevent Pb exposure is to recognize and eradicate sources of Pb; e.g., paint in old buildings, lead-contaminated water and soil, car and industries exhaust and some toys [53].

Pb may induce oxidative stress via reducing antioxidant power, ROS generation, GSH depletion, mitochondrial damage, and inflammatory responses. In a rat model study, increased MDA level, decreased GSH concentration and activities of SOD and GST were underlying pathways of Pb-induced oxidative stress which were attenuated by grape seed extract. Besides, glycogen synthase kinase-3β was involved in the progress of oxidative stress in rat kidney induced by Pb. GSK-3β phosphorylates and inhibits Nrf2 pathway, contributing to Pb-induced renal damage [54]. Pb exposure can also have effect on gene expression of IL-6 and TGF-β1 cytokines. This can happen along with activation of phosphoinositide 3-kinase and p38 mitogen activated protein kinase [55,56]. Consequently, in response to IL-6 and IL-1, production of factors associated with inflammation are stimulated. C-Reactive Protein (CRP), an acute phase protein, along with cyclooxygenase 2 (COX-2) and lipoxygenase, play roles in inflammation, which can cause damage to microorganisms and cellular structures [57].

#### 3.2.2 Acute lead poisoning

Lead poisoning, either acute or chronic, has been decreased during the past years due to improved vigilance of industrial and domestic exposures. However, it has not been eradicated completely. Although acute Pb toxicity is not common, it can still be induced following very high vapor inhalation, large ingestions and intravenous route. However, Pb-contained paint material and dust from old houses are considered the commonest sources of exposure. The clinical signs and symptoms usually consist of abdominal pain, hemolytic anemia, hepatic and pancreatic inflammations, and encephalopathy. When inhaled, Pb can cause cough, headache, confusion, drowsiness, metallic taste and even unconsciousness and convulsions. Eye contact leads to conjunctivitis [58].

Children have increased absorption ability of Pb and their classical manifestations mostly include neurological and GI symptoms and signs such as irritability, behavior and learning difficulties, abdominal pain, loss of appetite, vomiting and constipation. A body of evidence show that Pb affects the children's developing nervous system, thus, no safe blood lead concentration (BLC) is determined for children [58,59].

#### 3.2.3 Chronic lead poisoning

The majority of poisonings induced by Pb are chronic. It is a silent disease that can attack multiple organs. Chronic poisoning may be either subclinical or not severe enough to present definite or readily detectable manifestations, or it might have a range of different symptoms in the neurological, GI, hematological, renal and cardiovascular systems. Inorganic Pb is now classified as a probable human carcinogen (Group 2B) and a confirmed animal carcinogen [60].

#### 3.2.4 Prognosis for lead poisoning

The prognosis and outcome of lead poisoning are dependent on the duration and extent of exposure to this heavy metal. In children, lead poisoning can cause permanent health disorders, especially in the nervous system, that remains with them until adulthood. Higher levels of Pb in the blood can cause more severe and perhaps permanent damages; for instance, it can leave children with impaired intellectual ability and developmental problems [61]. Timely diagnosis and management of lead poisoning are critical since it can cause severe symptoms such as persistent GI and neurological disorders including encephalopathy [59]. Research have shown that exposure to Pb is associated with a higher chance of food allergies in adults.

#### 3.2.5 Severity classification (mild, moderate and severe)

The severity of toxicity can be classified as mild, moderate and severe. Mild poisoning usually has non-specific manifestations that can only be detected by careful examinations and a relative elevation in BLC (20–69 μg/dL). Central nervous system (CNS) findings such as mood swings and psychiatric problems, tiredness, sleepiness and irritability are most dominant. Physical examination is either normal or mild hypertension may be detected. In moderate poisoning when BLC reaches 70–100 μg/dL, clinical symptoms and signs typically include CNS and peripheral nervous system (PNS), including headache, loss of memory, GI manifestations such as abdominal pain, loss of appetite, constipation, as well as the involvement of many other organ systems [62,63]. Albeit rare in adults, acute encephalopathy is the hallmark of severe toxicity and is diagnosed by seizures, confusion, papilledema, optic neuritis and headaches. Further effects of severe Pb toxicity can be detected as peripheral neuropathy, anemia, colicky abdominal pain, impaired kidney function and cardiac dysrhythmias. In this stage, BLC usually reaches above 100 μg/dL.

#### 3.2.6 Common treatment of lead intoxication

Due to permanent and often life-threatening sequels of lead poisoning, timely treatment of symptomatic patients is critical. Irrespective of poisoning severity, the fundamental step of treatment is removing the lead sources or preventing further lead exposure whether occupational or non-occupational [51]. The next step is to determine the need for pharmacologic therapy with chelators and choose an appropriate method of treatment with respect to several factors such as clinical severity, BLC and each patient's individual factors. Currently, three chelating agents have been suggested for the treatment of lead poisoning; dimercaprol, calcium disodium ethylene diamine tetra acetic acid (CaNa_2_EDTA) and succimer. However, the only chelating agent that has U.S. FDA approval is succimer. It is used for the treatment of lead poisoning in children with BLC above 45 μg/dL. D-penicillamine, a chelating agent previously used for the treatment of lead poisoning, is now largely replaced by succimer, because of its significant adverse effects [64]. Overall, research has shown no proven benefit for administering chelators in patients with BLC 20–44 μg/dL [59].

#### 3.2.7 Recent advances in the treatment of lead intoxication

Despite stationary regulations, lead exposure and poisoning are still globally observed, because of the wide application and environmental existence of Pb. This has led to research into different treatment regimens to potentially improve the management strategies [65]. Human research on the treatment of lead poisoning with different severities have focused more on the currently available chelating agents by putting them into different mix and match regimens with or without natural substances [63,66] while experimental studies are more directed toward finding novel treatment options, especially botanical plants and herbal medicines [[67], [68], [69], [70], [71]]. A recent retrospective study on 79 male adults with chronic Pb poisoning, the efficacy of succimer, as the standard treatment, with that of D-penicillamine (750–1500 mg/day) combined with garlic tablets (1500–3000 mg/day) were compared. Subsequently, clinical and laboratory data (BLC) were collected and assessed. The results indicated that treatment with D-penicillamine plus garlic yielded the same outcome as succimer with a significantly lower cost. The price for 30 days of treatment with D-penicillamine plus garlic was reported to be 28.6 times less than that of a 19-day regimen with succimer [63]. Molavi et al. [72] attempted to compare the clinical and laboratory efficacy of succimer vs. D-penicillamine in patients with acute lead poisoning over a 5-year descriptive study. Total of 163 patients received any of the three types of treatments: D-penicillamine, succimer, and D-penicillamine plus succimer. Research data were collected once on admission and then in 2 weeks. Analyses of data depicted that there was similarity in clinical improvement among the groups, hence reporting D-penicillamine as an acceptable chelator for acute Pb poisoning. Similar to Vahabzadeh et al. [63], the outcome of the study performed by the Molavi research team confirmed the advantage of D-penicillamine when access to succimer is limited. Additional empirical evidence supports the efficacy of D-penicillamine as a proper alternative in the treatment of Pb poisoning when there is a shortage of other chelating agents [63,66,72].

Since Pb exerts part of its toxicity by inducing oxidative stress in different tissues, natural antioxidants have been the subject of interest in many studies. Usman et al. [71,71] compared the effect of Moringa oleifera (250 mg/kg) vs. ascorbic acid (50 mg/kg) on biochemical, hematological and histopathological changes in a model of subchronic lead toxicity in male rats. The agents were administered for 6 weeks, after which laboratory analyses were carried out. The results showed some improvements in Pb-induced changes in tissues. Another study [69] in rats with Pb-induced liver toxicity, Moringa oleifera was shown to have anti-anemic, immune stimulant and liver protective effects by inhibiting oxidative stress, inflammation and apoptosis. Mohammadi et. al [70]. evaluated the antioxidant and chelating potential of silibinin and nano-silibinin in rats poisoned with Pb. Rats were exposed to Pb (50 mg/kg) with or without different doses of silibinin and nano-silibinin (25, 50 and 100 mg/kg) for six days, and then blood samples for BLC and biochemical antioxidant tests were collected. Silibinin, and more potently its nano form, could prevent weight loss, decrease BLC and normalize malondialdehyde and nitric oxide (lipid peroxidation products) to standard levels. Both antioxidants were effective in preventing oxidative stress in Pb poisoning [70]. Silymarin acts as an antioxidant through different pathways, hence making it an attractive substance for a lot of toxicities and diseases. Another study in rats compared liver protective effects of silymarin and D-penicillamine in acute Pb poisoning. Rats were exposed to Pb (25 mg/kg), D-penicillamine (100 mg/kg) and silymarin (200 mg/kg). Pretreatment with silymarin and administration of D-penicillamine significantly decreased hepatic transaminases and improved liver antioxidant enzymes [73]. Garlic is another herbal substance that has been the subject of interest for many clinical investigations. Scientific evidence exists for noticeable effects of garlic on Pb poisoning when prescribed either alone or in combination with pharmaceutical chelator agents. It was shown that garlic has the potential to decrease Pb from blood and tissues as well as to possess antimicrobial, hypolipidemic, anticancer, antioxidant and antithrombotic properties [63,74,75]. When compared to D-penicillamine in the treatment of patients with chronic lead poisoning, garlic appeared safer and as efficacious as D-penicillamine. It was administered in the amount of 6 cubes of fresh herb in one study [75], and in the form of a 400-mg tablet in another [74], with similar promising results. El-Khishin et al. [76] also studied the therapeutic effect of garlic (20 mg/kg/day) combined with silymarin (1000 mg/kg/day) in comparison with those of DMSA (30 mg/kg/day) in a mice model of lead poisoning. Results showed comparable efficacy of garlic plus silymarin with DMSA in amelioration of Pb-induced nephrotoxicity and improvement of the histopathological patterns [76].

An animal research from India investigated the potential protective effect of Linum usitatissimum (Flaxseed) and Emblica Officinalis (Amla) on renal and hepatic poisoning of Pb [77]. Experimental rats were received doses of 100 mg/rat/day Emblica Officinalis or 300 mg/kg Linum usitatissimum orally for 45 days, and afterwards, oxidative stress enzymes along with histopathological analyses were carried out. Results revealed that Flaxseed and Amla had protective effects against Pb-induced hepatic and renal toxicity with relatively superior potency for the Emblica in comparison with the Flaxseed. The antioxidant effect of Flaxseed was also evaluated in another [78] study on Pb-poisoned rats that received daily doses equal to 100 mg/kg of Flaxseed dissolved in 1 mL of lemon juice for one month. The lemon juice and Flaxseed could improve kidney and liver function tests, prevent lipid peroxidation and inhibit the reduction of glutathione (GSH) in kidney tissues. The positive effects of Ginkgo biloba to alleviate lipid peroxidation induced by Pb are well documented in laboratory animals. When administered orally in doses of 50 and 100 mg/kg for 14 days, Ginkgo biloba could improve hematological parameters, the kidney and liver function indices, and show antioxidant activity in rats [79]. In addition, further evidence from rodent models of Pb toxicity suggests neuroprotective effects for Ginkgo biloba by which it can decrease neuropsychiatric symptoms in this poisoning [80,81]. Thymoquinone (TQ) as a natural bioactive compound has shown therapeutic effects for many diseases due to its antioxidant properties. In a rat model of subchronic toxicity, TQ was able to attenuate testicular and spermotoxicity due to Pb exposure [82]. A derivative of lysine amino acid (abbreviated as HTPL) was promising for alleviating the subchronic lead toxicity. HTPL was administered orally similar efficacy to those of succimer and EDTA [83].

Recent experimental investigations describe the ability of probiotic microorganisms, micronutrients (such as zinc and vitamin C), and some herbal extracts (such as tea polyphenols and grape seed extract) to effectively control Pb toxicity [84]. The supplements could reduce Pb accumulation in tissues, decrease oxidative stress and improve the hematological and neurological dysfunction in mice with insignificant adverse effects. A literature review on such probiotics reported that four types of microorganisms were most protective against Pb-induced toxicity in pre-clinical research: three bacteria (Lactobacillus sp., Pediococcus pentosaceus, Bacillus sp.) and one yeast (*Saccharomyces cerevisiae*) [67]. Therefore, along with standard chelating agents and other supportive treatments, dietary probiotic supplementation may be considered as a promising approach to manage Pb toxicity. Further herbal extracts and natural substances have been evaluated with promising outcomes in animal models of Pb toxicity. Cinnamon [85] and thymus vulgaris extract (thyme) [68] have also been indicated to possess favorable effect on the alleviation of kidney and liver toxicity of Pb via antioxidant and immune-stimulant properties. Further studies are required to better elucidate the application of herbal substances and nutrients in human beings.

Vit D has shown protective effects against Pb-induced tissue damage in an animal model. The co-administration of Vit D with Pb could mitigate tissue injuries induced by the toxic metal exposure. The role of antioxidant and anti-inflammatory mechanisms can be significant in such protective effect [86]. Glycine has a similar protective effect in Pb-induced organ toxicity based on another animal study [87]. NAC has shown mitigating effect on Pb toxicity in the testicular tissues of male rats. It was found that NAC treatment could improve sperm quality and morphological changes, and modulate antioxidant balance and apoptosis in testis tissue [88].

In conclusion, although The US FDA approved only succimer as an antidote for Pb poisoning, other chelating agents including CaNa_2_EDTA and D-penicillamine can be used, particularly when succimer is not available or its high cost will not be affordable. CaNa_2_EDTA should prescribed as infusion for severe cases, and D-penicillamine for mild to moderate cases in combination with garlic tablets. Other antioxidants may be used as preventive medications or in combination with the above-mentioned treatments.

### 3.3 Chromium (Cr)

Chromium (Cr) is used in several industry such as chrome plating, stainless steel, paint pigments, leather tanning, and some heat-resistant applications. Cr toxicity mainly results from the environmental and or occupational exposures [89]. However, the acute toxicity of Cr induces the multi-organ failure. The chronic Cr toxicity is more related to cancers [11,90,91]. Cr has various oxidation states of −2 to +6, however, the trivalent (Cr^3+^) and hexavalent (Cr^6+^) forms are more common and the most relevant to human exposures [11] (Table 1).

#### 3.3.1 Size of the problem and preventive measures against chromium exposure

Hierarchy of Controls is the main recommendation of National Institute for Occupational Safety and Health (NIOSH) for employers of Cr industries to consider the possible exposure for all staff and workers. For example; awareness of employers from Safety Data Sheet could reduce the Cr exposure and its hazard. The OSHA standards as average of 0.005 mg/m^3^ Cr (VI), 0.5 mg/m^3^ Cr (III), and 1.0 mg/m^3^ Cr (0) for an 8-h workday up to 40-h workweek. When employees expose more than 10 h per day, NIOSH recommends an exposure limit of 0.001 mg/m^3^ for Cr (VI) compounds in air. Air is another source of Cr exposure; people may be exposed to low levels of Cr by breathing its polluted air. The air of a city may be contaminated by Cr more than the rural or suburban air. In indoor air pollution by cigarette smoking, Cr concentration may reach to 10–400 times greater than the outdoor air concentrations. Thus reducing the smoking indoor air pollution could prevent the toxic effects of Cr. Agency for Toxic Substances and Disease Registry (ATSDR) suggests that total daily intake Cr from air should be less than 0.2–0.4 μg [92]. There is no recommendation for food or drug to reduce the absorption Cr or increase the Cr excretion. However, the oxidative stress may have an important role in reducing chronic Cr toxicity [11].

Markers of chromium (VI) exposure are generally biomarkers of oxidative stress, such as 8-hydroxy-2′-deoxyguanosine (8-OHdG), malondialdehyde (MDA), and glutathione (GSH). Other promising indicators have been proposed for dealing with chrome, and more data are needed to confirm their significance. Cr may induce oxidative stress via ROS generation, reducing antioxidant capacity, depletion of GSH, mitochondrial damage, and inflammation responses [93]. Xu et al. (2017) used oxidative stress parameters and DNA damage biomarkers to evaluate markers of Cr exposure 319 participants living in Cr-contaminated areas and 307 unexposed participants. They found that serum concentration of MDA, CAT, GSH-Px, and 8-OHdG were elevated in the exposed population compared to the control group [94]. Another study reported on the Club (Clara) cell protein (CC16) an immunosuppressive protein, surfactant-associated protein D (SP-D), tumor necrosis factor-α (TNF-α), and interleukin-6 (IL-6) as biomarkers for lung injury in blood of 91 Cr occupationally exposed individuals and 38 controls. The findings expressed that the levels of SP-D, TNF-α, and IL-6 were higher and associated with Cr presence at workplaces [95].

#### 3.3.2 Acute chromium poisoning

Cr^6+^ is a powerful oxidizing and caustic agent, thus massive Cr^6+^ ingestions can induce nausea, vomiting, diarrhea, vertigo, GI hemorrhage with or without bowel perforation, fever, muscle cramps, intravascular hemolysis with disseminated intravascular coagulation. Acute tubular necrosis leading to acute kidney injury, metabolic acidosis with elevated lactate concentration, hyperkalemia and uremia are consequences of acute massive exposure to Cr. Other presentations such as liver damage, acute multisystem organ failure, coma and even death were reported. Intoxicated patients are at high risk for acute respiratory distress syndrome (ARDS) up to 3 days. Cardiomyopathies and cardiovascular disease were reported after Cr inhalation [91]. Skin exposure to solutions contains Cr could induce penetrating lesions known as “chrome holes” or “chrome ulcers”, especially in injured epidermis. Exposure to 20 ppm Cr^6+^ may cause skin ulcers in unsensitized people. In severe dermal exposure, first and second degree burns that followed by delay onset GI and systemic symptoms may occur that resulted to death [96].

#### 3.3.3 Chronic chromium poisoning

The most route of chronic Cr exposure is by inhalation. Inhaled Cr^6+^ irritates the respiratory tract and induces inflammation. Continuous Cr inhalation results in inflammation and nasal septum perforation, chronic cough, shortness of breath, occupational asthma, bronchospasm, keratitis, gingivitis and periodontitis. Pneumoconiosis is also reported in chromite miners secondary to chronic deposition of chromium dust. Chronic inhalation of Cr^6+^ is associated with an increased risk of lung and nasal cavity cancer after exposure for one decade. The most common types of lung cancer are small cell and poorly differentiated carcinomas. However, inhalation of Cr^6+^ may be associated with nearly all pathologic types of lung cancer. Chronic Cr^6+^ exposure causes mild to moderate raise in the hepatic aminotransferase enzymes and urinary β_2_-microglobulin concentrations [97,98]. Some epidemiology studies and meta-analysis revealed the positive relationship between Cr^6+^ exposure and gastric cancer in human as well as animal studies. However, previous meta-analysis could not prove the relationship [[99], [100], [101]]. Dermal contact to Cr^6+^ induces two types of dermatological toxicities: skin ulcers and allergic contact dermatitis. Frequent exposure to compounds contained 4–25 ppm of Cr^6+^ induces sensitization and elicit chromium allergic contact dermatitis. About 5 % of cement workers who exposed to 10–20 ppm Cr^6+^ for some years present chromium sensitivity [102]. Chronic skin contact with Cr such as cement workers develops skin sensitivity, and one fifth of Cr workers complain of contact dermatitis. A 33 years old woman who chronically ingested 6–12 times of the recommended daily dose of over-the-counter chromium picolinate (Cr^3+^) for 4–5 months, presented with weight loss, anemia, thrombocytopenia and hemolysis. Her abnormal lab tests were raised aminotransferase enzymes 15–20 times normal, raised total bilirubin 3 times normal, raised serum creatinine to 5.3 mg/dL and blood urea nitrogen 152 mg/dL. The plasma Cr level was 2–3 times normal [103].

#### 3.3.4 Prognosis for chromium poisoning

The ingestion of high dose Cr (VI) compounds has poor prognosis and acute Cr poisoning could be fatal. The most frequent causes of death are large lesions in many tissues, GI hemorrhage and hypovolemic shock. If the patients survive, organ damage including acute liver and kidney failure may occur. Treatment is only symptomatic and using chelating agents have not been shown to be effective [104,105].

#### 3.3.5 Severity classification (mild, moderate and severe)

There is no classification for Cr intoxication. Severe exposures to Cr^6+^ compounds are usually intentional (suicide) or accidental, and are rarely occupational or environmental. The average human oral lethal dose of Cr^6+^ is estimated as 1–3 g.

#### 3.3.6 Common treatment of chromium intoxication

As for the other toxicologic emergencies, the basic supportive care of airway, breathing and circulatory should be done at the first step, and then decontamination shall be applied if required. Cr^3+^ compounds need limited decontamination with soap and water in case of skin contact. No pulmonary or GI decontamination is recommended [106]. In contrast, Cr^6+^ gastric lavage by nasogastric tube may be considered in a patient presents within a few hours after ingestion with no vomiting. However, nasogastric tube insertion is an aggressive procedure and may not be indicated in injured esophagus gastroscopy may be indicated. There is no recommendation for administration of activated charcoal. There are some case reports, that hemofiltration, hemodialysis and peritoneal dialysis were applied in Cr poisoning. Hemofiltration did efficiently remove Cr. Thus, it seems that peritoneal dialysis or hemodialysis is a reasonable therapeutic choice in the setting of acute or chronic kidney dysfunction due to Cr intoxication [107,108].

One of the main parts of metal poisoning treatment is chelating therapy. There are several suggested chelators such as BAL, EDTA, D-penicilamine, succimer and DMPS. However, there is little evidence for the effect of known chelators in Cr intoxication and there is no approved chelator for that [40,109]. EDTA was reported of not being effective for the management of Cr intoxication. Treatment of 16 patients with EDTA significantly raised the urinary excretion of lead, zinc, cadmium and calcium, but did not significantly change urine Cr level [110,111]. Anderson et al. [112] also reported an evaluated urinary Cr loss by various EDTA treatment regimes in two groups of human subjects who had taken Cr-supplementary. They found no relationship between urinary Cr and EDTA treatment [112]. Iron chelators have shown effective outcome in the treatment of Cr intoxication. Deferoxamine pretreatment in rat prevents nephrotoxicity by Cr^6+^ through lowering the oxidative stress or a chelating mechanism [113]. Iranmanesh et al. [114] evaluated the effect of single and a combination of deferasirox and deferiprone on Cr level of liver, kidney, intestine, spleen and testicle of chronic Cr^6+^ intoxicated rats. Rats received two doses of 15 and 30 mg/kg Cr^6+^ for 60 days. The Cr tissue levels of both groups were reduced by treatment with each of iron chelators, and combination of them was more potent. However, there is no evidence for these effects in humans [114].

#### 3.3.7 Recent advances in the treatment of chromium intoxication

Reactive oxygen species (ROS)-mediated oxidative stress is known as one important mechanism of Cr toxicity. Thus Cr-induced damages may be alleviated by agents that restore antioxidant balance [91]. NAC is a potent antioxidant that was applied in acetaminophen [115], lead [116], phosphine [117], paraquate [118], and THM poisoning [119]. Cr^6+^ induces oxidative stress and decreases GSH level. Thus, supplementary NAC could ameliorate the damage from Cr-induced oxidative stress [120]. Chelating is another suggested mechanism of antidotal effect of NAC against Cr. NAC could effectively excrete Cr through urine in potassium dichromate intoxicated rats. NAC also reversed oliguria in animal model. Moreover, NAC has also a chemoprotection mechanism by losing Cr^6+^ accumulation in cells through reduction of chromate to membrane-impermeable Cr^3+^ [121]. Increasing ROS, release of inflammatory cytokines and autophagy are important suggested mechanisms of skin adverse effects of Cr–induced allergic contact dermatitis. NAC can inhibit the expression of IL-1α and TNF-α mRNA induced by Cr (VI) in HaCaT cells [122]. Severity of intradermal and epicutaneous elicitation test of Cr-treated albino guinea pigs was reduced when the animals were treated by 1200 mg⁄kg⁄day of NAC. Sensitization rate of Cr hypersensitivity was also reduced by the same dose of NAC [123]. Pretreatment of Cr intoxicated HaCaT cells with NAC reduced apoptosis and autophagy and raised cell viability. Reduction in Cr cytotoxicity by NAC may be time dependent. Treatment of Cr intoxicated human proximal tubular epithelial cell line HK-2, with 600 or 1000 μg/mL NAC within 2 h reduced the expression of intracellular ROS and protected the kidney cell line from Cr^6+^-induced cytotoxicity. But 4 h and 8 h delayed treatment with NAC did not shown significant results [122]. NAC also improved the impaired glycemic state and enhanced insulin secretion and antioxidant competence in pancreatic beta-cells of Cr intoxicated rats [124].

A case study, reported a 47-year-old man who developed hepatic injury due to ingestion of 30 mL of plating solution. His liver function tests were improved after treatment by NAC and ascorbic acid. He was treated by 100 mg of intravenous ascorbic acid q8h and 1200 mg q12h orally NAC from 5th up to 13th day of hospitalization. However, his clinical conditions, general malaise, jaundice and total bilirubin level worsened again after discontinuing these therapies and then gradually improved [125]. Two cases of metal-on-metal hip-implanted patients treated by NAC were also reported. They were 67-year-old male and 81-year-old female who underwent Co/Cr hip implant and high Cr levels were detected in their serum. Serum Cr levels were reduced 87 % and 24 % after 300 mg/kg/day of NAC for 9 and 10 days, respectively [126]. Another case study reported an electroplating worker with severe Cr intoxication who developed multiorgan failure after skin exposure. His legs immersed in the electroplating solution that induced a chemical burn of 15 % of the body surface. He had been treated by intravenous DMPS 125 mg every 12 h, intravenous NAC 50 mg/kg every 4 h and ascorbic acid 100 mg every 12 h since the day after exposure. He also performed continuous veno-venous hemofiltration and plasmapheresis since 3rd day of hospitalization due to renal failure and thrombocytopenia. NAC, vitamin C, DMPS and plasmapheresis were discontinued at 10th day, however hemodialysis continued up to 30th day. He was completely recovered 3 months later [127].

Liver of chicken treated by Cr6+ showed increased malondialdehyde (MDA) content and decreased GSH content, Ca^2+^-ATPase activity, and mitochondrial membrane potential (MMP) level. Selenium supplementation could reverse Cr toxicity in chicken and alleviated Cr induced hepatic injuries in moderate dose [128]. In sub-acute and chronic animal models of Cr toxicity, melatonin; a powerful antioxidant; prevented hepatic enzymatic and non-enzymatic antioxidants reduction. Melatonin also reduced raised lipid peroxidation level and revealed liver histopathological changes [129]. The females first generation of rats; whom fed by their mother's milk and their mothers were treated by potassium dichromate through postpartum days 1–21; showed follicles atresia, raised cytochrome C and cleaved caspase-3, reduced antiapoptotic proteins, raised metabolic clearance of estradiol with reduced estradiol biosynthesis and impaired oxidant/antioxidant balance. Resveratrol (10 mg/kg body wt., through oral gavage daily), an antioxidant of grabs, showed a protective effect against toxic effects of Cr on ovary [130]. Membrane damage is considered an important mechanism of Cr cytotoxicity. Since alpha-tocopherol, a membrane protectant reduced this damage, it has been suggested that dietary supplementation of alpha-tocopherol may restrain the Cr-induced membrane damage [131].

Briefly, several promising approaches emerging from recent studies seems favorable in the treatment of Cr intoxication. NAC, selenium, melatonin, resveratrol, and alpha-tocopherol offer additional benefits for mitigating Cr-induced intoxication. Despite the encouraging findings, further clinical trials and mechanistic studies are essential to establish effectiveness of such treatment protocols.

### 3.4 Cadmium (Cd)

#### 3.4.1 Size of the problem and preventive measures against cadmium exposure

The major route of exposure to Cd is air, water, soil and food. Upon entering to the body, Cd exerts its toxic effects through different mechanisms [11]. It is emitted to the air from the weathering of rocks, forest fires and volcanic eruptions [132]. In addition, Cd accumulates in the environment resulting from industrial production including mining, smelting, battery manufacturing, fossil fuel combustion, Cd plating and chemical industry, fertilizer manufacturing and waste disposal [133,134].

Tobacco smoke is the major source of Cd atmospheric pollution and a serious health problem. The tobacco leaves contain one and two μg Cd in each gram dry weight of leaves. Therefore, each cigarette may have 0.5–1 μg Cd. The absorption rate of Cd from lung is much higher than the intestine. As a result, blood Cd concentrations may reach to 5 times higher in tobacco smokers in comparison with nonsmokers [135].

Apart from smokers and occupational population, food is the main environmental source of Cd exposure [[136], [137], [138]]. Cd pollution of aquatic environment is caused by release of pollutants from the air, metal smelters, coal combustion and the disposal of sewage sludge. In addition, atmospheric deposition, metal smelting, fuel combustion, sewage sludge and phosphate fertilizer are main sources of Cd contamination in soil. Cd may have long-term damaging effect on agricultural soils. Cd in the soil is transferred to plant and accumulates in agricultural products in large quantities. Because of elevated mobilization potential of Cd, it can easily enter the food chain.

The following strategies can be effective in controlling and reducing exposure to Cd and existing health hazards:

I. In the workplace with possible exposure with Cd, occupational hygiene and improved ventilation should be considered as the first line of defense.
II. The use of Cd and its compounds as stabilizers and pigments in plastics and paints or in packaging materials should be limited or banned.
III. Nickel-Cd batteries and accumulators containing with higher than allowable limit of Cd must be labelled and recycled or disposed properly.
IV. Production and smoking of cigarettes containing Cd must be limited.
V. Individuals should have adequate iron, zinc, and Se in the diet, because those who are at risk for micronutrients deficiency may absorb more oral Cd.
VI. The use of phosphate fertilizers in agriculture sector should be avoided.
VII. Every effort should be made to maintain the permissible level of Cd in air, drinking water, soil or food.
VIII. Natural and chemical substances can be applied to reduce the environmental Cd contamination. The seeds of some medical plants like peanuts (*Arachis hypogaea*) were suggested for water purification. Phytoremediation can also be used to extract and detoxify elemental pollutants in soil [139,140].

β-2-microglobulin (B2-MG) and N-acetyl-β-D-glucosaminidase (NAG) are well-established, sensitive, and the most common effect biomarkers to relate Cd or Cr exposure to renal tubular dysfunction. α-1-microglobulin in urine is another sensitive biomarker for Cd exposure implying proteinuria due to renal tubular damage [141,142].

#### 3.4.2 Acute cadmium poisoning

Acute Cd poisoning is characterized by respiratory irritation and occurs as result from inhalation exposure to Cd oxide. Within 6–12 h of exposure in a closed space, the patient manifests symptoms including cough, fever, and or respiratory distress or trouble breathing. Cd pneumonitis may progress to hypoxia, pulmonary insufficiency, or even death. Patients usually appear well and have a normal physical examination, oxygen levels, and chest X-ray on initial presentation. Cd pneumonitis can progress, with symptoms of rhonchi, impaired oxygenation and alveolar infiltrates on a chest radiograph. In severe cases, death often occurs within 3–5 days of exposure. Patients, who experienced pneumonia after an acute Cd poisoning, are at higher risk of pulmonary disorders. Generally, acute inhalation of Cd is more likely to occur than acute ingestion. Cd ingestion in high quantity causes symptoms like food poisoning.

#### 3.4.3 Chronic cadmium poisoning

##### 3.4.3.1 Nephrotoxicity

Cd accumulates predominantly in the kidneys. The hallmark feature of renal damage in chronic Cd toxicity is proteinuria that is generally irreversible even after exposure removal. Other early signs of injury may include glomerular dysfunction, calcium loss and tubular lesion. In severe cases, glycosuria, hypercalciuria, hypophosphaturia, polyuria aminoaciduria, and decreased buffering capacity may be manifested [139,143].

##### 3.4.3.2 Pulmonary Toxicity

The incidence and/or severity of Cd pulmonary toxicity depend on dose and duration of exposure. Some evidence indicated on a possible involvement of Cd in lung injury such as chronic obstructive disease and emphysema. Improvement in pulmonary symptoms has been reported after removal from Cd exposure [143].

##### 3.4.3.3 Musculoskeletal Toxicity

Environmental Cd exposure can be a cause of osteotoxic effect. Age, sex, cumulative dosing and route of exposure are the reason of appearance of osteomalacia in *Itai-itai* victims (old women) and in patients with environmental exposures. The *Itai-Itai* patients experienced a broad range of signs and symptoms, such as a low bone mineralization, bone decalcification, a high incidence of fractures and distortion of the long bones, intense bone pain, osteomalacia, and osteoporosis. Cd exposure may promote the development of musculoskeletal health problems, such as osteoarthritis and rheumatoid arthritis [144].

##### 3.4.3.4 Other Organ systems

Cd poisoning can cause nerve damage to the olfactory nerve and cortical function and lead to development of Parkinsonism [145,146]. Cd exposure is also associated with increased risk of atherosclerosis and heart failure [147]. Indeed, Cd can cause a deficiency of testosterone production leading cell hyperplasia [148]. Hepatotoxic and immunosuppressive effects are not prominent features in humans with Cd exposure.

##### 3.4.3.5 Carcinogenicity and Cancer burden

Cd compounds are categorized as carcinogenic and recognized as a carcinogen in humans by different regulatory agencies. The International Agency for Research on Cancer (IARC) classified Cd as Group 1, carcinogenic to humans [149]. Epidemiological surveys suggest that occupational Cd exposure is associated with an increased risk of lung cancer. Some reports suggested that Cd might be involved in prostate, genitourinary, breast and renal cancer, although this linkage is weaker than for lung cancer. Furthermore, Cd has also been linked to malignancies of liver, pancreas, stomach, thyroid and hematopoietic system [143,144,150,151].

#### 3.4.4 Prognosis for cadmium poisoning

The prognosis for Cd toxicity depends on many factors including route of exposure, dose, and duration of exposure, rapid detection of Cd in blood samples, severity of clinical manifestation and initial management of poisoning. Patients with severe intoxication may need more aggressive treatment.

In acute Cd poisoning, death may occur due to respiratory irritation following inhalation exposure. The inhalation exposure to 5 mg/m^3^ for 8 h may be lethal. The ingestion lethal dose is estimated in the range of upward from 150 mg.

In chronic exposure, various organs can be affected including kidney, lung, musculoskeletal and nervous system. Cd exposure can be associated with early signs of renal dysfunction, proteinuria, calcium loss and tubular lesion. Cd has a long half-life in the kidney and therefore it would be reversed if it is detected in lower body burden. In patients with pneumonia, aggressive treatment should be considered to prevent of persistent restrictive lung disease [152].

#### 3.4.5 Severity classification (mild, moderate and severe)

There is no classification for severity of Cd toxicity. Severe toxicity can occur through accidental or intentional (suicide) poisoning [143,153]. The lowest human's lethal dose of Cd is estimated as near 70–80 mg/kg.

#### 3.4.6 Common treatment of cadmium intoxication

Immediate considerations focus on GI decontamination and supportive care. Acute poisoning due to large ingestion of Cd compounds is rare, but it may be fatal. If vomiting has not occurred spontaneously, gastric lavage is recommended soon. Pulmonary damage is suspected in patients with exposure to Cd fumes. Corticosteroids are used to reduce the inflammation and control the respiratory complications. All patients with evidence consistent with Cd exposure should be hospitalized and evaluated for renal, hepatic, GI, urinary and respiratory tracts injury and function [144]. Hemodialysis is not effective in treatment of Cd intoxication and it is indicated if the renal failure is present [143].

Although using chelators is the preferred medical treatment in acute ingestion of Cd, the benefit of chelation therapy remains unproven. Among different chelating agents, succimer can be assumed as an effective drug due to decreasing the GI absorption without accumulation in target organ and improve survival. Other chelators with possible benefits include DMPS, diethylenetriaminepentaacetic acid (DTPA). Although dimercaprol (BAL) is a powerful chelator for Cd as other THM metals, it is reported that the Cd-BAL complex is much more nephrotoxic than Cd itself [154]. Thus, dimercaprol increases the Cd of liver and kidney and it is not recommended to give in Cd intoxication [143]. It has been reported that the other chelators could be either ineffective or detrimental as for penicillamine, tripolyphosphate, nitrilotriacetic acid, CaNa_2_EDTA and dithiocarbamates [155].

#### 3.4.7 Recent advances in the treatment of cadmium intoxication

Variety of preventive and treatment approaches based on underlying mechanisms against Cd toxic effects have been studied in recent years. Natural compounds with antioxidant and anti-inflammatory properties such as curcumin, ginger, resveratrol, physalis extract, grapefruit juice, allicin, royal jelly, spirulina, quercetin, caffeic acid phenethyl ester, vit C, vit E, NAC, coenzyme Q10, alpha-tocopherol, selenium and zinc may be good candidates to reduce Cd poisoning [[156], [157], [158], [159]]. N-benzyl-D-glucamine dithiocarbamate (BGD), N-tetramethylene dithiocarbamate (ATC) and N-p-hydroxymethylbenzyl-D-glucamine dithiocarbamate (HBGD) have shown protective effect against Cd cytotoxicity by decreasing the Cd level (increasing the urinary excretion) and oxygen species activity [[160], [161], [162]]. Royal jelly from worker honey bees, showed protective effect against Cd-induced nephrotoxicity in mice. The observed reno-protective effect makes this natural compound a potent candidate for renal protection against Cd toxicity [163]. A combination therapy of DMSA combined with its analog MiADMSA, which is not yet commercially available, has been claimed to mobilize intracellular Cd deposits to some extent via its ability to cross biomembranes [164].

The results of *in vitro* and *in vivo* studies indicated that the probiotic microorganisms are able to protect against acute and chronic Cd toxicity mainly by reduction of the intestinal Cd absorption; decreasing the Cd accumulation in the tissue and alleviating the oxidative stress [165]. It also has been reported that essential trace elements such as zinc, selenium, or iron can be used for the susceptible population to long-term exposure to Cd [155].

In conclusion, the treatment of Cd intoxication is an evolving field, with various natural compounds and chelating agents showing promise in mitigating Cd toxicity. Natural antioxidants like curcumin, ginger, and royal jelly, along with established chelators such as DMSA, show viable therapeutic options. Furthermore, the role of probiotics and essential trace elements in reducing Cd exposure highlights the complex approach necessary for effective management. Future research should focus on clinical trials to validate the efficacy of these treatments.

### 3.5 Arsenic (As)

#### 3.5.1 Size of the problem and preventive measures against arsenic exposure

The Environmental Protection Agency (EPA) and WHO set the safe contaminant level of As in drinking water to be less than 10 ppb (0.010 mg/L) [166,167]. In the workplace, the limit set by the OHSA is 10 μg (μg) of As per cubic meter of air for 8-h shifts and 40-h weeks. One of the strategies for prevention of exposure to As is providing a safe water supply for drinking, irrigation of food crops and food preparation. As contaminated soils are remediated by mycoremediation, amalgamating various bioremediation techniques and phytoremediation [168,169]. Also, human may be exposed through food with pesticides residues grown in As rich soil or irrigated with contaminated water. There may be As in some foods, including meat, poultry, fish, and rice. Rice being the food for many people, significantly contributes more As than any other food source. One of the prevention techniques is the adaptation of cooking and irrigation ways for rice. Using genetically modified rice that would accumulate less As within the rice grains is another strategy [[170], [171], [172]]. Nutritional status may play a role in preventing As toxicity. Diet rich in selenium, zinc and other antioxidants (such as vitamin E, vitamin C), and folate help to decrease As toxicity. Research on the potential protective roles of thiol and selenol compounds, including new chelating agents against As toxicity are of great importance in the future [[173], [174], [175], [176], [177]].

Oxidative damage markers for lipids such as malondialdehyde (MDA) as main lipid peroxidation product, proteins (e.g., carbonylation), and nucleic acids such as 8-hydroxydeoxyguanosine provide a more accurate representation of oxidative stress and its biological implications due to As induced damage. As can induce both eustress and distress conditions in many organs and tissues [178,179].

#### 3.5.2 Acute arsenic poisoning

Arsenic has two chemical valences of three and five. The three-valence compound (As_2_O_3_) is more toxic than the five-valence compounds. There are variations in different people, population groups and geographic areas for As exposure [11,180,181].

##### 3.5.2.1 Acute toxicity

Acute As poisoning occurs more commonly from accidental ingestion, homicide or suicide and infrequently from the workplace. Acute toxicity presents with gastroenteritis followed by hypotension. Gastroenteritis manifestations include nausea, vomiting, diarrhea, abdominal pain, garlic odor in breath and body tissues, hypersalivation, thirst, dehydration, and dysphagia. Symptoms begin within 30 min to 2 h after ingestion and resolve within 12 h, but may present for days after exposure. The *cholera-like* or *rice water* diarrhea is the classic type of diarrhea which may also be bloody. Arsenate (pentavalent form of As) is corrosive and may cause oral burns, dysphagia and GI bleeding [180,181]. Hypotension secondary to dehydration and volume loss is the most important cardiovascular manifestations. Electrocardiogram (ECG) abnormalities may be observed hours to days after exposure including prolonged QT interval, QRS lengthening, non-specific ST-segment changes, T wave flattening, torsade de pointes and ventricular fibrillation [182]. Neurologic symptoms including delirium, tremor, headache, weakness, leg/muscular cramps, encephalopathy, hyperpyrexia, seizures, autonomic neuropathy with anhidrosis, sweating, flushing and unstable blood pressure have been reported [11]. Pulmonary involvement may present as cough, dyspnea, and chest pain which is more commonly seen with inhalation exposure. In severe cases, non-cardiogenic pulmonary edema, adult respiratory distress syndrome and acute respiratory failure can be present. Proteinuria, glycosuria, leukocyturia, hematuria, and acute tubular necrosis with oliguria have been reported hours to days after exposure. Hepatic involvement including elevated liver enzymes, jaundice, pancreatitis, fatty infiltration, congestion, central necrosis, cholangitis, and cholecystitis; and hematologic abnormality such as anemia, thrombocytopenia, leukopenia, bone marrow suppression, acute hemolysis and disseminated intravascular coagulation are another presentation of acute poisoning. Other reported manifestations include skin rashes, rhabdomyolysis and conjunctivitis [11]. As can cross the placenta and is teratogenic. Fetal demise due to the toxic As concentrations in the fetal organs has been reported [183]. Patients with acute toxicity should be monitored for at least four to 6 h after suspected ingestion. Spot urinary As concentration; As levels in 24-h urine; chest x-ray and plain abdominal X-ray is requested for patients with acute poisoning. Organic As compounds present in the urine may be from food sources. Therefore, the patient should be asked if shellfish was recently eaten. Hair samples may be positive 30 h post exposure. They cannot differentiate between ingestion and external exposure. Serum As levels are not a reliable marker for As toxicity because of rapid As clearance from the bloodstream [[184], [185], [186]].

Arsine gas (AsH_3_) is an extremely toxic form of As compounds. Inhalation of arsine gas above 10 ppm is lethal. Arsine gas is colorless and odorless that does not cause tissue irritation. It is a hemolytic agent. The symptoms appear very soon after exposure. Hemolysis with hematuria and jaundice can last for several days [180,187,188]. Symptoms include garlic breath odor, nausea, and vomiting, abdominal pain, diarrhea, headache, chest pain, imbalance, tachycardia, fever, hematuria, liver function disturbance. Renal failure 1–12 h after exposure up to 24 h after acute exposure may be observed. Port wine colored urine, reddish conjunctiva, and jaundice reported up to 48 h after exposure. High peaked t-waves, conduction abnormality, heart blocks, and asystole may be observed. Cardiac and renal failures are commonly the causes of As-induced death [181,187,188].

##### 3.5.2.2 Subacute toxicity (1–3 weeks)

Survivors of acute As poisoning and patients who are slowly intoxicated environmentally may manifest sub-acute toxicity. One to three weeks after exposure to As, some clinical manifestations may occur as following:

Neurologic disorders are initially sensory which may begin 2–4 weeks after resolution of the initial signs of acute As poisoning. Weakness, loss of deep tendon reflexes, gait disturbances, temperature sensation, vibratory sensation and quadriplegia reported in severe cases, and in rare instances, respiratory muscle insufficiency. There are also reports of headaches, confusion, memory loss, seizures and delirium. Leukopenia, thrombocytopenia, basophilic stippling and anemia, hypotension, hemolytic vomiting, diarrhea, abdominal pain, difficulty in swallowing, severe hypovolemia, cardiomyopathy, ventricular dysrhythmias and congestive heart failure are other clinical manifestations [180,181].

#### 3.5.3 Chronic arsenic poisoning

Chronic As toxicity is associated with various clinical manifestations known as arsenicosis [167]. Peripheral neuropathy and skin lesions are the most common effects of chronic As poisoning. ([[189], [190], [191], [192], [193]], Wang, Karvonen-Gutierrez et al., 2022, [194]). Skin lesions are hyperpigmentation on the face, neck and back and a “raindrop” appearance on the trunk and neck; hyperkeratosis of the palms of the hands and soles of the feet. Skin manifestations are not commonly seen in As inhalation exposures. Mees lines as 1–2 mm wide transverse white bands across the fingernails presents 4–6 weeks after exposure. Dermatitis, melanosis, vesiculation warts, alopecia, conjunctivitis and corneal ulceration are other manifestations [167,192]. Peripheral neuropathy, motor paralysis, specifically of the distal extremities, hearing loss, encephalopathy, symmetrical peripheral polyneuropathy, muscle fasciculation and ataxia are the other manifestations of chronic toxicity [167]. Cardiovasuclar toxicity includes carotid atherosclerosis, hypertension, arrhythmias, pericarditis, Raynaud's syndrome, acrocyanosis (intermittent), ischemic heart disease, black foot disease, and cerebral infarction due to microcirculation abnormalities [167,191]. GI effects may be observed as anorexia, abdominal pain, diarrhea, weight loss, esophagitis, gastritis, and colitis. Liver toxicity present with increased hepatic enzymes with enlarged and tender liver, jaundice, cirrhosis, fatty degeneration, portal hypertension without cirrhosis, ascites and angiosarcoma. Anemia, leukopenia, thrombocytopenia due to bone marrow hypoplasia, impaired folate metabolism and karyorrhexis are hematologic features. As might influence the risk of metabolic syndrome in midlife women [195]. It is also related to low working memory in adolescents [196,197]. As has been classified as group 1 human carcinogens and the related toxicity may lead to carcinogenicity and mutagenicity. It elevates the risk of the skin, kidney, bladder, liver, lung and breast cancer [189,193].

#### 3.5.4 Prognosis for arsenic poisoning

Prognosis of As poisoning correlate to the toxicity severity which depends on combination of several factors including age, As state, route of exposure, dose, duration of exposure, underlying diseases, early detection of As in biological samples and the availability of chelators. In acute As poisoning, death is usually due to hypovolemic shock and cardiovascular collapse. The minimal lethal dose is in the range of 1–3 mg/kg. The estimated lethal exposure dose of inorganic As is 0.6 mg/kg. Lethal doses can result in death within 1–4 days after ingestion [181,198]. Survivors of an acute poisoning will mostly develop neural injury (sensory and motor disturbances). They may have been cardiac, renal, liver and skin problems and the prognosis may be poor. Recovery from chronic As poisoning may take long time and may not be completed especially for peripheral neuropathy [181,198].

#### 3.5.5 Severity classification (mild, moderate and severe)

There is no specific severity classification for As toxicity. Toxicity can be acute, sub-acute and chronic. Severity of poisoning depends on several factors including the type of As compound, valence state, toxicity dose, route of exposure, duration of exposure and underlying diseases. Other factors include individual variations in methylation and excretion [11].

#### 3.5.6 Common treatment of arsenic intoxication

##### 3.5.6.1 Acute As poisoning

Aggressive intravenous fluid and electrolyte replacement therapy is lifesaving in severe poisoning. However, maintaining the fluid balance is important because of cerebral and pulmonary edema which may be occurring. GI decontamination is controversial. Although As is poorly adsorbed to activated charcoal, it is performed because of low likelihood of harming the patient. It is also administered in the case of co-ingestion. In case of radiopaque-material visualization in the GI tract, bowel irrigation is recommended until it is no longer visualized on repeated radiographs. Continuing nasogastric suction removes As re-secreted in the gastric or biliary tract [199]. Hemodialysis should be a consideration for patients with acute kidney injury (AKI), oliguria and or renal failure. Also, washing with soap and water and rinsing affected skin, antiemetic, blood transfusion (in case of hemolysis), using mineral supplements that lower the risk of potentially fatal heart rhythm problems are other modalities. Treatment with magnesium sulfate, amiodarone, lidocaine and defibrillation if ventricular tachycardia or torsades de point occurs, is suggested. The use of IA, IC and III antiarrhythmics secondary to risk QT prolongation should be avoided. Seizures are treated with benzodiazepine and general anesthesia if necessary [199,200]. Succimer, BAL and DMPS are the most important used chelators. The goal of chelation therapy is to achieve a 24-h urinary As level below 50 μg/L. The chelators are most effective within minutes to hours post exposure [[201], [202], [203]].

##### 3.5.6.2 Arsine gas poisoning

Treatment primarily involves supportive care and is aimed at preserving renal function. Red cell transfusion, folate or iron supplementation is necessary for the patients with significant hemolysis. Hemodialysis should be considered for patients with oliguria, AKI or renal failure. Exchange transfusion removes non-dialyzable arsine ([199], Wang, Karvonen-Gutierrez et al., 2022).

##### 3.5.6.3 Chronic As poisoning

Treatment is mostly symptomatic. It involves primarily on the identification and elimination of the toxic source as well as to provide supportive measures. Stoppage of drinking of As-contaminated water is the mainstay of the management. Workers who are exposed occupationally to As may express their concerns with employers. After an intervention, follow-up urine and clinical testing may be applied to verify a reduction in exposure [199,200]. Chelation has limited efficacy in chronic exposures. In cases of sub-acute and chronic toxicity, it is recommended to await laboratory confirmation prior to beginning chelation therapy, unless there is a worsening of the patient's clinical condition. Succimer is the preferred chelator for sub-acute and chronic toxicity. Liver function tests and metal level assessment should be monitored in patients needing prolonged treatment.

#### 3.5.7 Recent advances in the treatment of arsenic intoxication

An analog of succimer, monoisoamyl ester, have increased survival in rats poisoned by As [204,205]. A chelating agent monoisoamyl 2,3-dimercaptosuccinic acid (monoisoamyl DMSA, MiADMSA) is being currently in clinical phase I study for the treatment of chronic As poisoning [206]. Increased antioxidant capacity of cells could reduce As toxicity. Montelukast was effective at reducing As-induced epithelial-mesenchymal transition in bronchial epithelial cells [207]. An animal model revealed that NAC has a protective effect against As-induced liver injury [207]. Melatonin had also a significant effect in reducing age-dependent cytotoxicity of As in rats' brain [208]. In an *in vitro* study, green tea (Camellia sinensis) showed a preventive effect in As oxidant stress [209,210]. Polyphenol-rich apple (Malus domestica) peel extract ameliorate the cardiotoxicity of As trioxide in h9c2 cells [211]. Spirulina (Cyanobacteria) extract removed As from isolated liver cells [212]. Moreover, virgin coconut oil (VCO) protected hepato-renal damage against As-induced oxidative stress [213]. The antagonism effect of ginger against As-induced hepato-renal poisoning has been evaluated in a study by Panda et al. [214]. Ginger powder effectively counteracted the toxic effects of As in ducks [214]. Kumar et al. [215] revealed the protective efficacy of Coriandrum sativum seeds extract against toxicity of sodium arsenite in mice [215]. Coriandrum sativum seeds extract had ameliorative effect against liver and kidney toxicity induced by As. In another study, Aspalathus linearis ameliorated tissue toxicity associated with As exposure in rats [216]. *In vivo* studies have been shown that Viscum album and Phyllanthus emblica leaves, Syzygium cumini leaves, Moringa oleifera leaves and Ipomea aquatica aerial parts had ameliorative effects in As toxicity. Flavonolignan (silibinin), Allium sativum, Curcuma longa, Silybum marianum and some fibers and algae were the effective herbals for the treatment of As intoxication [217]. The intake of zinc [218,219] and selenium [[220], [221], [222]] may reduce As-induced toxicity. An inverse relationship has been shown between dietary intake of folate [[223], [224], [225], [226]], pyridoxine and riboflavin [227], vitamins A [228], C [174,229,230] and E [[231], [232], [233]] with the severity of skin lesions in As toxicity. However, another study did not find a statistically significant relationship between the supplementation with vitamin E, selenium, or both and the severity of skin lesions [234]. Inter-individual susceptibility plays an important role in As toxicity. Variation in As biotransformation, genetic, environmental and physiological factors including lifestyle and nutritional status, other diseases, and co-exposure toward other THM may regulate the extent of As-induced skin lesion [235,236]. Clinical trial studies have shown the usefulness of vitamins A, C and E in arsenicosis patients [237]. Nutritional supplementation is controversial in the absence of dietary deficiency. More researches are needed. Natural compounds including β-carotene, α-tocopherol, genistein, α-LA, eriodictyol, hydroxytyrosol, epigallocatechin-3-gallate, oleuropein, ellagic acid, rutin, curcumin, quercetin, naringenin, sulforaphane, allicin, lutein, biochanin, resveratrol and D-pinitol have been alleviated As toxicity in some animal models and *in vitro* studies [217]. Garlic oil has been effective in the keratosis induced by As [238]. Co-administration of phytochemicals with a chelating agent eliminated more As from the body with less adverse effects because of a reduced dose of chelating agent [239]. Chelating agents such as MiADMSA when administered along with curcumin as a phytochemical or herbal extract have been shown to be effective [240]. It has been suggested that nutritional combination therapy is more beneficial for the treatment of the chronic As exposure than usual chelation monotherapy. However, clinical study is needed to accurately determine the dose. Proteins, amino acids, flavonoids, polyphenols, jiggery containing polyphenols, vitamin C, carotene and other biologically active components, fruits and honey are the supplements that can be useful in the management of As toxicity [217]. Ayurvedic therapies are also areas explored against As toxicity. However, the drug safety regulations are required [241,242]. Because As contamination have a big impact on human health, there are still challenges in the field of new treatment of the metal toxicity [243].

## 4 General discussion and comparison of the treatment between the five THM

The five THM are different in terms of application, toxicity and the target organs effects. However, there is similarity on their treatment. Following diagnosis of intoxication by the THM (Table 2), particularly in acute forms, general treatment usually starts by gastric aspiration and lavage and a saline laxative, soon after referral if clinically indicated. This might prevent further absorption of further amounts of the THM.

**Table 2:** Clinical evaluating tools to diagnose and determine intoxication by THM.

Medical history and physical examination	Ask about symptoms, occupational and other exposures, do a physical examination
Hematological and biochemical lab tests	Finding abnormal biochemical and hematological indices related to THM toxicity
THM blood and serum test	Measurement of THM level in whole blood or in serum
Complete blood count (CBC)	Finding abnormal blood indices related to THM toxicity
U/A	Urine evaluation for detection or quantification of THM
Hair, nail and saliva level of THM	Tissue evaluation for detection or quantification of THM
Electrocardiogram	ECG abnormalities may be observed hours to days after exposure
X-ray and CT scan imaging tests	Provide data of occurrence and location of abnormalities related to THM toxicity

**Fig. 1:**
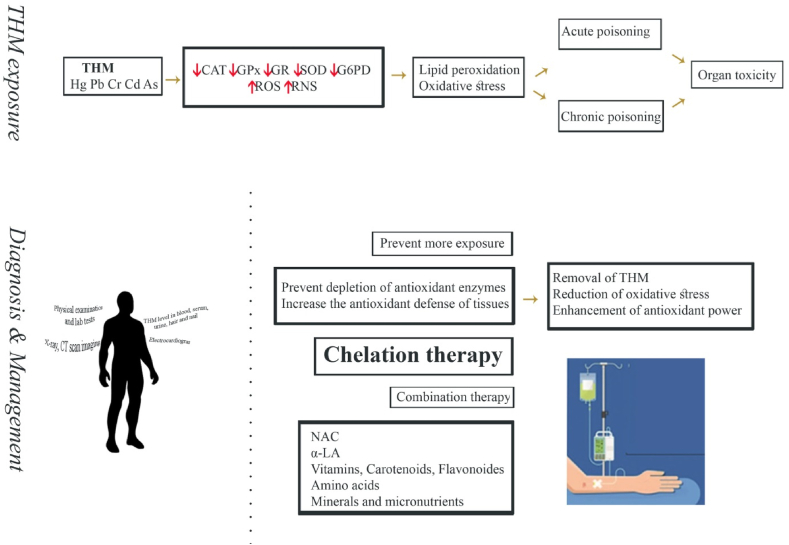
Exposure, diagnosis and clinical management of intoxication by THM; THM: toxic heavy metal.

**Fig. 2:**
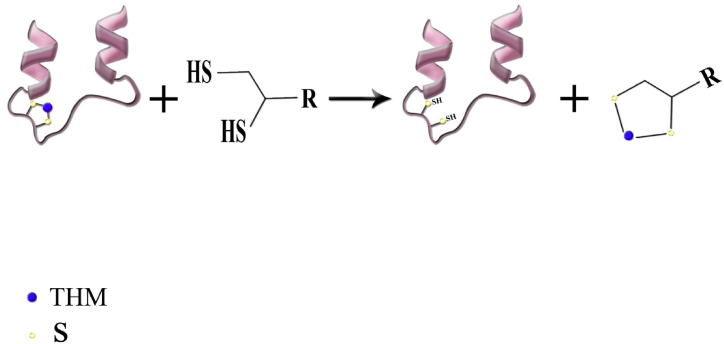
Simple schematic expression of chelation therapy: Release of a toxic metal-bounded protein from THM by using chelating agents; THM: toxic heavy metal; R is used to represent the rest of the molecular structure of the chelating agent.

Some of the toxic metals require special attention. For example, brain edema management in lead poisoning, treatment of hemolytic reaction for arsenic and therapy for exudative dermatitis in chromium poisoning should be done. The use of chelating agents as THM antidotes is well known and research for the management of these THM poisonings using new chelators is ongoing. Along with the antidote therapy, particular attention on the target organs such as the CNS, kidneys and liver should be taken to reduce the organ damage. Fig. 1 briefly demonstrates the diagnose and clinical management of THM poisoning. Fig. 2 shows the schematic of chelation therapy for the clinical management of intoxication by the THM.

**Fig. 3:**
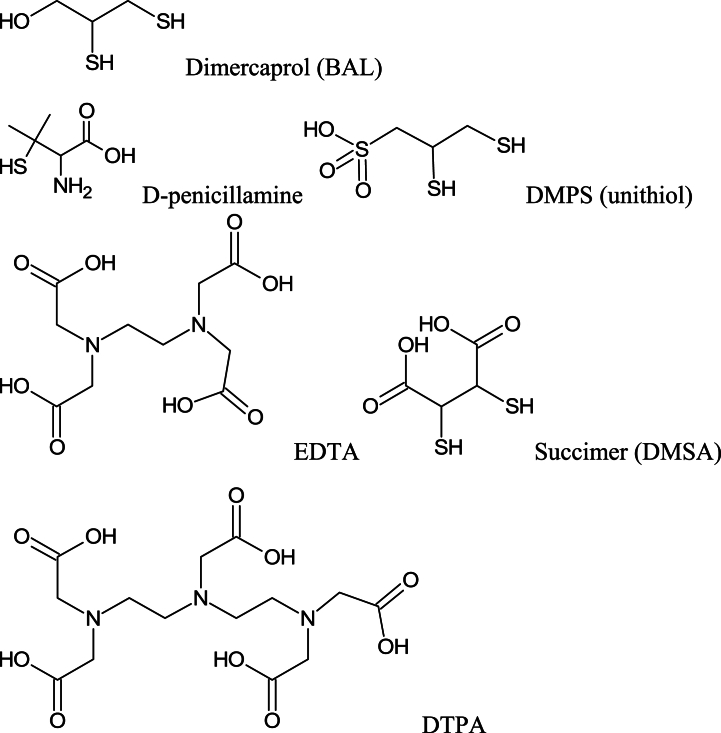
Structure of suggested chelators for the clinical management of the THM poisoning.

Chelating agents are the compounds which allow attachment of two or more donor atoms to the THM. This causes the formation of a stable complex in the form of a ring structure. Chelating agents are capable of effectively mobilizing THM deposits in the urine. Structure of suggested chelating agents used for the clinical management of the THM poisoning is shown in Fig. 3. There are potential risks and side effects associated with such treatments. For instance, chelating agents may cause renal toxicity and imbalances in electrolytes. One study identified renal adverse effects including proteinuria, increased serum creatinine, and the risk for Fanconi syndrome. The renal dysfunction was resolved upon discontinuation of the medicine [244]. Inadequate mobilization of THM metals from tissues represents another drawback of chelation therapy. Certain chelators, particularly hydrophilic ones like DMSA are effective in enhancing renal excretion but struggle to access and mobilize metals stored intracellularly. Consequently, this can result in incomplete detoxification of THM metals from the body [245].

Due to many drawbacks in the application of commonly used chelating agents, their administration in the clinical management of THM has come with limitations. Having sides effects which sometimes would be serious (Table 3), the loss of essential metals, redistribution of THM, inability to remove THM from intracellular compartments can be mentioned as such drawbacks. Constant endeavors to replace suggested known chelators with newer and less complicated ones or concomitant therapy of known chelating agents with newer compounds have attracted much more attention. Research aiming to find new chelating agents continued and has been reported during recent years. Recent studies indicate that the clinical management of poisoning by the THM with different severities has basically focused on the commonly available chelators by putting them into different regimen of therapies.

**Table 3:** Suggested known chelators for the clinical management of the THM poisoning.

Chelator	Mercury (Hg)	Lead (Pb)	Chromium (Cr)	Cadmium (Cd)	Arsenic (As)	Best Route of Administration	Side Effects/Considerations
Dimercaprol (BAL)	For acute inorganic Hg/contraindicated for organic Hg/limited efficacy in chronic poisoning	Suggested	Suggested	Cd-BAL complex is much more nephrotoxic than Cd itself	Limited efficacy in chronic poisoning	IM	Injection site pain, nausea, vomiting, fever, kidney toxicity, headache, restlessness, increased blood pressure, tachycardia
CaNa_2_EDTA	Suggested	Suggested	Non-optimal effectiveness	Non-optimal effectiveness		IV	Nausea, vomiting, fever, increased blood pressure, arthralgia, allergic reactions, local inflammation, nephrotoxicity, headache, anorexia, myalgia, fatigue, thirst, chills, cardiac complications
D- penicillamine	For acute inorganic mercury	Significant adverse effects; currently replaced by succimer	Suggested	Ineffective or detrimental	Suggested	PO	Allergic reactions, immunodeficiency, worsening of neurological manifestations in patients with Wilson's disease
Succimer (DMSA)	For acute inorganic mercury	FDA-approved	Suggested	Suggested	Suggested: chelator of choice	PO	Nausea, vomiting, diarrhea, fever, hives, dizziness, weakness
Unithiol (DMPS)	For acute inorganic mercury		Suggested		Limited efficacy in chronic poisoning	PO, IV	Rash, nausea, leucopenia
DTPA				Suggested		IV	

IM: intramuscular injection, IV: intravenous injection, PO: oral administration.

Combination therapy aimed to use medications to act by different mechanism of actions. If the prescribed drugs act through a synergism action, if will enhance the efficacy of treatment. One might do combination therapy with known and newer chelators, while another will have the strategy of known chelating agent plus antioxidants [246]. Different treatment regimens were recommended mainly with application of natural thiol-containing substances which may have chelating properties for THM. Garlic is a natural compound containing thiol and has shown promising results in the treatment of lead poisoning. Some other investigations have used compounds with known antioxidant, anti-inflammatory, antiapoptotic and anti-cancer properties for the management of THM poisoning (Table 4). Such compounds include natural, synthetic or semi-synthetic substances that have undergone animal studies and the beneficial results of their usage were reported. Phenolic, flavonoid and tannin compounds which are enriched in fruits, show protective effects, are the sources of the reducing antioxidant power. On the other hand, experimental research is more directed towards finding novel treatment options, especially from botanical plants and herbal medicines. Some reports on animal studies provided important findings on the protective effects of the antioxidant and anti-inflammatory ingredients present in the natural products against THM poisoning [86,87,163,247]. In this regard, interest has been taken with reference to nano specifications of the compounds in order to gain higher therapeutic efficacy [70,240,248].

**Table 4:** Frequently used supplements for the treatment of THM poisoning in recent literature.

THM	Recently reported supplements
Hg	Shenkang injection, Calcium carbonate, garlic tablet and N-acetyl cysteine (NAC), Selenium supplementation = Selenomethionine and sodium selenite, α-LA, Flavonoids = Myricetin
Pb	D-penicillamine plus garlic, Moringa oleifera, ascorbic acid, silibinin and nano-silibinin, Silymarin, garlic, garlic combined with silymarin, Linum usitatissimum (Flaxseed) and Emblica Officinalis (Amla), lemon juice and Flaxseed, Ginkgo biloba, probiotic microorganisms [bacteria (Lactobacillus sp., Pediococcus pentosaceus, Bacillus sp.) and yeast (*Saccharomyces cerevisiae*)], micronutrients (such as zinc and vitamin C), herbal extracts (such as tea polyphenols and grape seed extract), Cinnamon and thymus vulgaris extract (thyme), Vit D, Glycine, NAC, TQ
Cr	NAC, ascorbic acid and NAC, vitamin C, Selenium supplementation, melatonin, Resveratrol, alpha-tocopherol, zinc
Cd	curcumin, ginger, resveratrol, physalis extract, grapefruit juice, allicin, royal jelly, spirulina, quercetin, caffeic acid phenethyl ester, vit C, vit E, NAC, coenzyme Q10, alpha-tocopherol, selenium and zinc, N-benzyl-D-glucamine dithiocarbamate (BGD), N-tetramethylene dithiocarbamate (ATC) and N-p-hydroxymethylbenzyl-D-glucamine dithiocarbamate (HBGD), Royal jelly, probiotic microorganisms, zinc, selenium, or iron
As	monoisoamyl 2,3-dimercaptosuccinic acid (monoisoamyl DMSA, MiADMSA), NAC, melatonin, green tea (Camellia sinensis), Malus domestica, Spirulina (Cyanobacteria), virgin coconut oil (VCO), ginger, Coriandrum sativum, Aspalathus linearis, Viscum album and Phyllanthus emblica leaves, Syzygium cumini leaves, Moringa oleifera leaves and Ipomea aquatica aerial parts, Flavonolignan (silibinin), Allium sativum, Curcuma longa, Silybum marianum, some herbal fibers and algae, vitamins A, C and E, zinc and selenium, folate, pyridoxine and riboflavin, β-carotene, α-tocopherol, genistein, α-LA, eriodictyol, hydroxytyrosol, epigallocatechin-3-gallate, oleuropein, ellagic acid, rutin, curcumin, quercetin, naringenin, sulforaphane, allicin, lutein, biochanin, resveratrol and D-pinitol, garlic, MiADMSA with curcumin, amino acids, flavonoids, polyphenols, jiggery, Ayurvedic therapies

Table 3 presents the comparison of suggested known chelating agents that are commonly administered for the management of the five THM. Briefly, the following points were summarized:

I. In order to prevent the loss of important nutritional metals such as calcium, iron, zinc during chelation therapies, monitoring of these elements in the urine are required.
II. Adjusting the dose of chelators is a function of the serum level of the THM and the patient's symptoms and signs of toxicities.
III. There is no specific antidote for Hg, but chelation therapy is favored in patients with moderate to severe intoxication.
IV. Limited efficacy in chronic organic Hg poisoning has been shown for dimercaprol (BAL). BAL also redistributes this THM to the brain and is therefore contraindicated.
V. Succimer has U.S. FDA approval for the clinical management of plumbism.
VI. There are few evidences on the effect of known chelators in Cr intoxication. However, NAC and ascorbic acid have shown beneficial results either alone or in combination with the standard therapies.
VII. Chelation therapy remains unproven for medical treatment of acute Cd poisoning. As for succimer, DMPS and DTPA are assumed to be effective chelating therapies.
VIII. Cd-BAL complex has shown more nephrotoxic effects than Cd itself.
IX. Nutritional combination therapy has shown more efficacious in clinical management of chronic As toxicity.
X. There is no antidote for arsine gas poisoning. Clinical management is mainly supportive and no chelation therapy is warranted.
XI. Chelation therapy has limited efficacy in chronic As toxicity.
XII. Administration of dimercaprol should be done at least 4 h before the CaNa2EDTA in Pb poisoning.
XIII. Although suggested for the management of Cr intoxication, EDTA has limited efficacy because of high removal of beneficial minerals such as zinc and calcium.
XIV. Apart from lead poisoning, succimer may also be used in Hg or As toxicities.

Treatment of THM poisonings using different chelating agents may have the same healing results. For example, a treatment course of D-penicillamine plus garlic can have the same outcome as succimer does, but at a lower cost [74]. It seems that such chelator of choice is suitable when succimer is not available or the high cost will not be affordable.

Phytochemicals can provide protection against free radicals’ injury and help to maintain human health. For this reason, the use of phytochemicals together with conventional chelating agents has been considered. Curcumin, as an antioxidant and anti-inflammatory medication that has received much attention in recent years, has been shown to be effective in the removal of THM from the body [240]. Quercetin is commonly found in substantial quantities in plants, and possesses potent antioxidant properties. Quercetin can scavenge free radicals and chelate metals. The dietary antioxidant quercetin seems promising to attenuate the toxicity of environmental THM exposure. The catechol group in the B-ring may be responsible for metal chelation and in oxidation processes [249,250]. Quercetin administration during chelation therapy with MiADMSA shows beneficial effects on the protection of inhibited blood δ-aminolevulinic acid dehydratase (ALAD) activity and depletion of As level from target organs in mice [251]. Another animal study revealed that allicin and quercetin, alone or in combination, can improve the antioxidant potent of the liver and alleviate Pb-induced liver injury and apoptosis. The protection resulted through the PI3K signaling pathway and stronger effects achieved by their combination [252]. Similarly in a mice study on testicular germ cells, quercetin attenuated Cd-induced cell apoptosis by suppressing oxidative stress mediators and also via downregulation of the expression of Bax and caspase-3 and upregulation of Bcl-XL expression [253]. Some of these phytochemicals have not yet been tested in a randomized clinical trial. However, these medications could not be prescribed as a main treatment, but may be used as adjunct therapy.

## 5 Conclusion

Chelation therapy has been regarded as a promising medical intervention for THM poisonings. Its application for the management of THM poisoning is an important aspect of heavy metals detoxification that was reviewed. Succimer is a less toxic metal chelator in chelating agents’ family. It has the FDA approval for the clinical management of plumbism. Similarly, Dimercapto-propanesulfonic acid has also fewer side effects. The two are currently gaining increased acceptance among clinical toxicologists. However, there is no specific antidote for mercury poisoning. Dimercaprol is almost no longer being used as an antidote of choice in the treatment of chronic THM poisoning. Comparison of clinical management of the toxicity by the five heavy metals reveals similar treatment strategies to prevent more exposure, using chelating agents and to improve the antioxidant power. On the other hand, some of them require specific interventions to reduce the toxicity. Findings of many studies conducted in the recent years indicate that combination therapy using two chelating agents or co-administration of natural/synthetic antioxidants along with chelators is beneficial in getting more pronounced therapeutic efficacies.

Because of drawbacks in the application of commonly known chelating agents, treatment with bioactive compounds which have antioxidant and anti-inflammatory properties has been the subject of much interest in recent research. However, despite the promising results observed, clinical trials on their therapeutic benefits need to be established to determine the efficacy and safety in humans. Development of less toxic chelating agents has still very high research priority. Moreover, the development of orally administrable chelating agents for home health care would likely be of great interest for future research.

### 5.1 Limitations

Despite the recent advances in the development of clinical management of heavy metals intoxication, several limitations can be mentioned. Firstly, there is often a lack of comprehensive clinical trials that adequately assess the safety and efficacy of these novel therapies across diverse populations. Many studies are carried out in controlled settings, which may not accurately reflect real-world situations in which patients show different levels of exposure and underlying diseases. Additionally, the long-term effects and potential side effects of some treatments remain poorly understood, which requires further investigation. Variation in individual responses is another limitation to the treatments, which is affected by genetic, environmental, and health factors. Furthermore, this study deals with the treatment of poisoning of heavy metals in general and does not cover special human populations such as children, pregnant women, and individuals with underlying diseases. Lastly, while this review highlights several promising therapies, the accessibility and cost of these treatments in different regions may hold back their widespread implementation, especially in low-resource settings.

## CRediT authorship contribution statement

**Mahdi Balali-Mood:** Writing – review & editing, Supervision, Methodology, Conceptualization. **Nastaran Eizadi-Mood:** Writing – original draft, Investigation, Formal analysis, Data curation. **Hossein Hassanian Moghaddam:** Writing – original draft, Investigation, Formal analysis, Data curation. **Leila Etemad:** Writing – original draft, Investigation, Formal analysis, Data curation. **Mohammad Moshiri:** Writing – original draft, Investigation, Formal analysis, Data curation. **Maryam Vahabzadeh:** Writing – original draft, Investigation, Formal analysis, Data curation. **Mahmood Sadeghi:** Writing – review & editing, Writing – original draft, Methodology, Investigation, Formal analysis, Data curation, Conceptualization.

## Data availability

The data that support the findings of this study are outlined in the current review either as tables, figures, and context references.

## Funding

The authors gained no financial support regarding the preparation of this manuscript.

## Declaration of competing Interest

The authors declare that they have no known competing financial interests or personal relationships that could have appeared to influence the work reported in this paper.

## Glossary

AKI: Acute kidney injury
α-LA: alpha-lipoic acid
ALAD: δ-aminolevulinic acid dehydratase
APL: Acute promyelocytic leukemia
ARDS: Acute respiratory distress syndrome
ATC: N-tetramethylene dithiocarbamate
ATSDR: Agency for Toxic Substances and Disease Registry
BAL: British Anti-Lewisite
BBB: Blood brain barrier
BGD: N-benzyl-D-glucamine dithiocarbamate
BLC: Blood lead concentration
CaNa_2_EDTA: Calcium disodium ethylene diamine tetra acetic acid
CdTe: Cd telluride
CKD: Chronic kidney disease
CNS: Central nervous system
DMPS: 2,3-dimercaptopropane sulfonate
DMSA: Dimercaptosuccinic acid (succimer)
DTPA: Diethylenetriaminepentaacetic acid
ECG (EKG): Electrocardiogram
EPA: Environmental Protection Agency
FDA: Food and Drug Administration
GI: Gastrointestinal
GSH: Glutathione
HBGD: N-p-hydroxymethylbenzyl-D-glucamine dithiocarbamate
IARC: International Agency for Research on Cancer
ICU: Intensive care unit
MDA: Malondialdehyde
MiADMSA: Monoisoamyl 2,3-dimercaptosuccinic acid
MMP: Mitochondrial membrane potential
NAC: N-acetylcysteine
NIOSH: National Institute for Occupational Safety and Health
OSHA: Occupational Safety and Health Administration
PEL: Permissible exposure limit
PNS: Peripheral nervous system
ROS: Reactive oxygen species
VEGF: Vascular endothelial growth factor
VCO: Virgin coconut oil
WHO: World Health Organization
